# Returning to school after a terror attack: a longitudinal study of school functioning and health in terror-exposed youth

**DOI:** 10.1007/s00787-018-1196-y

**Published:** 2018-07-12

**Authors:** Lise Eilin Stene, Jon-Håkon Schultz, Grete Dyb

**Affiliations:** 1 Norwegian Centre for Violence and Traumatic Stress Studies, NKVTS, Oslo, Norway; 2 Department of Education, University of Tromsø, The Arctic University of Norway, Tromsø, Norway; 3 Institute of Clinical Medicine, Faculty of Medicine, University of Oslo, Oslo, Norway

**Keywords:** Stress disorders, Posttraumatic, Terrorism, Academic performance, Adolescent, Young adult, Quality of life, Public health, Social support, Education, Mental health services

## Abstract

Terrorist attacks and mass shootings often involve youth. Knowledge is needed on how this may impact their health and functioning. This study investigates perceived academic performance and school wellbeing in 237 terror-exposed survivors of the Utøya youth camp attack according to their sociodemographic characteristics, health and mental health service (MHS) utilization. Semi-structured interviews were conducted after 4–5 and 14–15 months. The year following the attack, 143 (61%) survivors reported impaired academic performance and 66 (29%) impaired school wellbeing. Female survivors more often reported impaired performance. Non-Norwegian origin, being financially disadvantaged and less social support were associated with impaired wellbeing. Sleep problems, posttraumatic stress, anxiety/depression, somatic symptoms, and lower life satisfaction were associated with both impaired performance and impaired wellbeing. Survivors who had received MHS were more likely to report impaired or improved academic performance and school wellbeing. Higher age and posttraumatic stress reactions were associated with impaired academic performance after multivariate logistic regression adjustments for gender, somatic symptoms and social support. When additionally adjusting for impaired school wellbeing, age and impaired wellbeing were associated with impaired performance. Only posttraumatic stress reactions were associated with impaired wellbeing after similar adjustments. Non-Norwegian origin and being financially disadvantaged were not significantly associated with impaired wellbeing after adjusting for posttraumatic stress reactions, age and gender. Our findings demonstrate how a terrorist attack can considerably deteriorate young survivors’ performance and wellbeing at school, which is associated with poorer health. Consequently, it is important to provide appropriate school support, and coordinate MHS with follow-up at school.

**Electronic supplementary material:**

The online version of this article (10.1007/s00787-018-1196-y) contains supplementary material, which is available to authorized users.

## Introduction

Terrorist attacks and shooting sprees often involve adolescents and young adults. Trauma exposure may be particularly detrimental in the transition from adolescence to adulthood as it may perturb the young survivor’s psychosocial development and education with potential long-term adversity, including chronic mental disorders and disability at school and work [1, 2]. Traumatic experiences may activate posttraumatic stress responses that disturb sleep, concentration and cognition, and consequently undermine the school functioning [3–8]. Injuries and somatic symptoms, such as chronic pain and fatigue, might further impair concentration and lead to absence from school [9]. A supportive school environment may be an important source of social support, which is considered among the most protective factors against posttraumatic mental health problems [10]. Conversely, negative reactions from social surroundings augment the risk of mental health problems [11]. Establishing effective preventative measures is especially important in youth as it may prevent many years lost to disability [12]. Schools and educational institutions may play a crucial role in the follow-up of youth exposed to traumatic events. These are places where deterioration of health and functioning can potentially be uncovered and prevented, and interventions implemented [13]. Although terror attacks and mass shootings frequently affect school pupils and students, little is known about their academic performance and their wellbeing at school after such events. To strengthen educational institutions’ and health care services’ responses to mass casualties, and prevent long-term disability and marginalization, knowledge is needed about the relationship between young survivors’ school functioning, posttraumatic health and mental health service utilization.

This study examined how youth perceived their school functioning the year following exposure to a terrorist attack. On July 22, 2011, a solitary extreme right perpetrator carried out two attacks in Norway. After detonating a bomb in the Oslo Government Quarter, he executed a shooting massacre at the summer camp of a political youth organization on the Utøya island. During the 1.5-h-long shooting, 69 persons were killed, and many were injured or risked drowning while trying to escape by swimming. The Utøya shooting is considered a severe trauma because of the high number of fatalities and physically injured, that many lost close ones, the young age of those involved and the fact that they were designated targets.

The survivors were mostly students in upper secondary school or higher education programs. The school semester started 4 weeks after the massacre, and the Norwegian Ministry of Education instructed the schools to contact all students to plan their return to school and to tailor possible adaptations throughout the school year [14, 15]. In addition, detailed information was posted on the Ministry’s website about pupils’ rights to educational adaptations, including information from the Norwegian Education Act (2006) on permitted absence and alternative ways of assigning grades and completing classes in high school when the student’s absence is high [2, 16]. Teachers and school health workers were asked to be proactive and provide survivors with close follow-ups, supporting them to complete their school program. The importance for the school to work together with health professionals was frequently mentioned in the Ministry’s communication.

The aim of this study was to generate knowledge to improve educational institutions’ and health care services’ support for young survivors of terror attacks and other mass traumas, and prevent impairments in their school functioning and health. Our specific objectives were to (a) examine the perceived academic performance and school wellbeing in youth exposed to mass trauma and their need for school support, and (b) investigate their perceived school functioning according to their sociodemographic characteristics, health status and specialized mental health service utilization.

## Methods

### Participants and procedures

This study includes data from two survey waves of the survivors of the Utøya attack. Overall, 495 survivors who had been on the Utøya island during the shooting were identified from police records. The recruitment consisted of three stages: (1) a postal invitation, (2) a telephone call, and (3) an interview with those who answered the call and agreed to participate. Four survivors aged ≤ 13 years and one who lived abroad were excluded, hence postal study invitations were sent to 490 survivors. Semi-structured face-to-face interviews were performed at 4–5 months (wave 1) and 14–15 months (wave 2) after the attack. The study had an open cohort design in which all of the eligible survivors were invited to participate at both waves. Overall, 325 (66%) of the survivors participated at wave 1, and 285 (58%) at wave 2 [17]. At the second survey wave, there was a set of questions exclusively for survivors who were school pupils or students during the school year following the shooting (2011/2012). In total, 237 survivors answered at least one of these questions and were included in this study.

### Variables

*School functioning* was assessed at both waves by two questions concerning (1) the survivors’ perceived academic performance, and (2) their wellbeing at school. At wave 1 they were asked during the interview, and at wave 2 in a questionnaire filled out directly after the interview, with the interviewer available in case of questions. At wave 2, survivors were asked if they had been school pupils or students during the school year following the shooting (2011/2012). Those who answered yes were asked if they completed the year (yes/no), if their academic performance or wellbeing at school/studies had changed (two separate questions with three response alternatives: worse/unchanged/better), and if they had received auxiliary school support (yes/no) after the shooting. In the analyses, the response alternatives “unchanged” and “better” were merged into one category (not impaired). Furthermore, they were asked whether they had needed practical facilitations (e.g. adjournments of test/exams), auxiliary academic assistance from teachers, additional social or emotional support, and how satisfied they were with school support. These four questions had five response alternatives: (a) not at all, (b) to a small extent, (c) to some extent, (d) to a large extent, and (e) to a very large extent. The alternatives were collapsed into three categories: no/little (a + b), some (c), and much/very much (d + e).

*Mental health service (MHS) utilization* (yes/no) was assessed at wave 1 (covered contact with mental health services approximately 0–5 months after the attack) and wave 2 (covered contact from January 1, 2012 until interview at wave 2, i.e., approximately 5–15 months after the attack). *Predisaster MHS utilization* was assessed at wave 2 by a question on whether they had received MHS before the terror attack.

*Posttraumatic stress reactions* in the past month were measured by the University of California at Los Angeles PTSD Reaction Index (UCLA PTSD-RI) [18]. The total score covers 17 items that correspond to the 17 PTSD DSM–IV symptoms rated on a 5-point Likert scale from 0 = never to 4 = most of the time. Three items have two alternative formulations where the item with the highest score is included. Reactions experienced “much of the time” and “most of the time” were defined as clinical symptoms. Cronbach’s alpha was 0.89 both at wave 1 and 2.

*Symptoms of depression and anxiety* were assessed with Hopkins Symptom Checklist-8 (SCL-8). It comprises eight items scored from 1 (not bothered) to 4 (very much bothered) which cover symptoms of depression and anxiety the past 2 weeks [19, 20]. Cronbach’s alpha was 0.85 at wave 1 and 0.89 at wave 2. *Somatic symptoms* the preceding 2 weeks were assessed by a short eight-item version of Children’s Somatic Symptoms Inventory (CSSI-8) [21]. It covers pain in stomach, head, lower back, and arms/legs, faintness/dizziness, rapid heartbeat, nausea/stomach problems, and weakness. Each item is scored from 1 (not bothered) to 4 (very much bothered). Cronbach’s alpha was 0.77 at wave 1 and 0.75 at wave 2. *Social support* was measured by seven items scored from 1 (much less than I would like) to 5 (as much as I would like) from the Duke-University of North Carolina Functional Social Support Questionnaire (FSSQ) [22]. Cronbach’s alpha was 0.80 at wave 1 and 0.85 at wave 2. *Sleep problems* were assessed by a question from the UCLA PTSD-RI on how often during the past month they had trouble going to sleep or waking up often during the night using five response alternatives. Survivors who answered twice a week or more were classified as having sleep problems. *Terror exposure* was assessed at wave 1 by a sum score of 13 potentially traumatic events occurring during the attack, which has been shown to be independently associated with mental health problems [23]. Participants at wave 2 who did not participate at wave 1 answered questions on terror exposure in wave 2. *Life satisfaction* was evaluated at wave 2 by the question “How satisfied are you with your life, in general?” scored from a scale from 1 (very dissatisfied) to 10 (very satisfied).

*Financial status* was evaluated by asking survivors how they perceived their parents’ (survivors who lived with their parents) or their own (survivors who did not live with their parents) economy compared to others. It was evaluated at wave 1 except for participants who joined the study at wave 2. In the latter case, the financial status was evaluated at wave 2. The five response alternatives were dichotomized into financially disadvantaged (much or somewhat poorer) or not (similar, somewhat better, and much better).

*Age* was assessed at the time of the attack as a continuous variable with one decimal. Furthermore, *non*-*Norwegian origin* was defined as having both parents born abroad.

### Ethics

The study participation was based on written informed consent. Parental consent was required for survivors younger than 16 years of age, as stipulated by Norwegian laws. The interviewers had a health-related background and were trained in conducting research interviews with traumatized individuals at a seminary before the study. They worked in teams of two, and after each wave there was a seminary where they could share experiences. The interviewers were instructed to assist survivors in contacting suitable services if they identified survivors with unmet help needs. A phone line was provided for the interviewers where they could receive support. The study was approved by the Regional Committees for Medical and Health Research Ethics South East and North.

### Statistical analysis

In the bivariate analyses, Pearson Chi-square tests were used for categorical variables, and independent *t* tests for continuous variables. Our level of statistical significance was *p *<0.05. The two dependent variables; academic performance and school wellbeing at 14–15 months after the attack (wave 2), were dichotomised into impaired or not by merging the response categories “unchanged” and “better” into “not impaired”. Due to sample strength considerations (statistical power), we limited the number of degrees of freedom to five in our multivariable analyses. We did an à priori selection of the following independent variables: age, gender, and posttraumatic stress reactions; somatic symptoms; and social support measured 4–5 months after the attack (wave 1). Furthermore, we did a post hoc analysis to examine whether non-Norwegian origin and being financially disadvantaged would remain significantly associated with impaired school wellbeing after adjustments for age, gender and posttraumatic stress reactions. The percentages were calculated from the total number of responses for the respective variables, and the analyses were based on the total number of answers. We lacked wave 1 data from 26 survivors who participated at wave 2 only. Otherwise, there was little missing data. No respondents had more than two items with missing values in any of the scales (PTSD-RI, SCL-8, CSSI-8 and FSSQ-7). The mean scores of the answered items were used in the analyses. The crude and adjusted odds ratios (ORs) were presented with 95% confidence intervals (CI). The analyses were effectuated with IBM SPSS version 24.

## Results

**Fig. 1:**
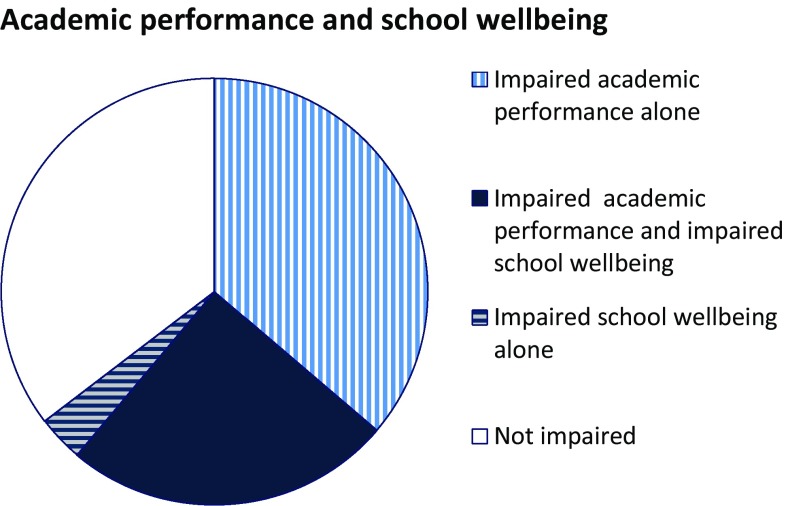
Overall 229 survivors of the Utøya attack answered both questions on academic performance and school wellbeing 14–15 months after the attack, including 57 (25%) who reported both impaired academic performance and impaired school wellbeing, 83 (36%) impaired performance alone, 8 (4%) impaired wellbeing alone, and 81 (35%) neither impaired performance nor impaired wellbeing

The median age of the student participants at the time of the attack was 17, 3 years (range 13.3–33.8 years, 95% were 23.1 years or younger), 113/237 (48%) were female, and 19/233 (8%) were of non-Norwegian origin. The characteristics of terror exposure among the participants are described in Table 1. At 4–5 months after the attack (wave 1), 135 (69%) survivors perceived their academic performance to be impaired, 44 (23%) to be unchanged and 16 (8%) to be improved compared to before the attack. Next, 14–15 months after the attack (wave 2), 143 (61%) survivors reported their academic performance to be impaired, 61 (26%) to be unchanged, and 29 (12%) to be improved (Fig. 1). Among survivors who disclosed impaired academic performance at wave 1, 100 also did so at wave 2; whereas 19 reported unchanged, and 15 improved academic performance. With respect to impaired school wellbeing, 58 (30%) survivors disclosed impaired, 51 (26%) improved, and 85 (44%) no changes in wellbeing 4–5 months after the attack; whereas 66 (29%) survivors disclosed impaired, 49 (21%) improved, and 116 (50%) reported no changes in wellbeing 14–15 months after the attack. Of those who reported impaired wellbeing at wave 1, 33 also did so at wave 2, whereas 19 of them reported unchanged and 5 improved wellbeing at wave 2. Overall, 61 (26%) reported that they did not complete the study year. In the bivariate analyses, impaired academic performance reported 14–15 months after the attack was associated with female gender; sleep problems; symptoms of posttraumatic stress, anxiety and depression; somatic symptoms; lower life satisfaction, and not completing the study year (Table 2). Impaired wellbeing was associated with non-Norwegian origin; being financially disadvantaged; sleep problems; symptoms of posttraumatic stress, anxiety and depression; somatic symptoms; less social support; lower life satisfaction; and not completing the study year. The great majority of the survivors with impaired wellbeing at wave 2 also reported impaired performance, *n *=57 (88%). Furthermore, survivors who reported impaired performance and wellbeing were less likely to be satisfied with the school’s support measures.

**Table 1:** Characteristics of exposure in the sample of student survivors of the Utøya attack

Exposure characteristics	*n* (%)
Saw the terrorist or heard his voice, *n *= 232	164 (70.7)
Hid from or ran from the terrorist, *n *= 232	226 (97.4)
Heard gun shots	233 (100)
Heard people screaming, *n *= 231	215 (93.1)
Smelled gunfire or other distinct smells, *n *= 228	74 (32.5)
Saw someone be injured or killed, *n *= 231	147 (63.6)
Heard someone be injured or killed, *n *= 230	184 (80.0)
Saw dead bodies, *n *= 231	198 (85.7)
Touched dead bodies or injured people, *n *= 232	102 (44.0)
Was afraid of being seriously injured, *n *= 231	174 (75.3)
Was afraid that he/she would die, *n *= 231	181 (78.4)
Saw the terrorist point the gun at him/her or realized that he had shot at him/her, *n *= 233	100 (42.9)
Was afraid that he/she would drown, *n *= 232	67 (28.9)
Felt threatened by the police, *n *= 227	93 (41.0)

All survivors (100%) heard gun shots; hence a sum of exposure (0–13) was calculated from all characteristics except “heard gun shots”

**Table 2:** Characteristics of survivors of the Utøya attack by impaired school performance and impaired wellbeing assessed at wave 2, 14–15 months after the attack

Characteristics	School functioning year after attack					
	Impaired performance (*n *= 233)		*p* value	Impaired wellbeing (*n *= 231)		*p* value
	Yes (*n *= 143)	No (*n *= 90)		Yes (*n *= 66)	No (*n *= 165)	
	*n*/mean (%/sd)	*n*/mean (%/sd)		*n*/mean (%/sd)	*n*/mean (%/sd)	
Mean age at attack (*n *= 233)	18.45 (2.78)	18.10 (2.10)	0.299	18.20 (2.88)	18.31 (2.39)	0.768
Female gender (*n *= 233)	75 (52)	35 (39)	0.044	36 (55)	72 (44)	0.133
Non-Norwegian origin (*n *= 229)	14 (10)	5 (6)	0.274	10 (15)	9 (6)	0.016
Financially disadvantaged (*n *= 229)	32 (23)	14 (16)	0.168	20 (31)	25 (15)	0.007
Admitted to hospital after attack (*n *= 233)	11 (8)	6 (7)	0.769	5 (8)	12 (7)	0.936
Predisaster MHS utilization (*n *= 234)	35 (25)	22 (25)	0.986	19 (29)	38 (23)	0.352
Sleep problems						
Wave 1 (*n *= 208)	63 (50)	16 (20)	< 0.001	34 (61)	43 (29)	< 0.001
Wave 2 (*n *= 231)	43 (30)	12 (14)	0.004	23 (35)	30 (18)	0.008
Impaired performance after 4–5 months						
Wave 1 (*n *= 193)	100 (86)	34 (44)	< 0.001	47 (92)	86 (61)	< 0.001
Impaired wellbeing after 4–5 months						
Wave 1 (*n *= 192)	44 (39)	13 (17)	0.001	33 (65)	24 (17)	< 0.001
Received auxiliary school support (*n *= 230)	100 (73)	48 (53)	0.002	49 (78)	100 (61)	0.019
Satisfied with study support (*n *= 197)						
No/little	26 (22)	8 (11)	0.047	17 (32)	17 (13)	0.001
Somewhat	28 (23)	11 (16)		15 (28)	23 (17)	
Much/very much	66 (55)	51 (73)		22 (41)	94 (70)	
Did not complete study year (*n *= 229)	50 (36)	9 (10)	< 0.001	28 (43)	30 (19)	< 0.001
Mean exposure (0–13) (*n *= 229)	8.37 (2.30)	8.20 (2.17)	0.584	8.92 (2.13)	8.10 (2.26)	0.013
Posttraumatic stress reactions (mean PTSD-RI)						
Wave 1 (*n *= 209)	1.65 (0.71)	1.28 (0.68)	< 0.001	1.95 (0.68)	1.34 (0.67)	< 0.001
Wave 2 (*n *= 233)	1.32 (0.64)	1.04 (0.64)	0.001	1.66 (0.63)	1.03 (0.58)	< 0.001
Symptoms of anxiety/depression (mean HSCL-8)						
Wave 1 (*n *= 209)	2.16 (0.64)	1.79 (0.57)	<0.001	2.44 (0.60)	1.86 (0.58)	< 0.001
Wave 2 (*n *= 233)	1.86 (0.63)	1.59 (0.57)	0.001	2.19 (0.68)	1.59 (0.46)	< 0.001
Somatic symptoms (mean CSSI-8)						
Wave 1 (*n *= 209)	1.81 (0.56)	1.54 (0.45)	< 0.001	1.97 (0.62)	1.59 (0.46)	< 0.001
Wave 2 (*n *= 233)	1.68 (0.48)	1.50 (0.45)	0.005	1.84 (0.54)	1.51 (0.41)	< 0.001
Social support (mean FSSQ-7)						
Wave 1 (*n *= 209)	4.51 (0.61)	4.62 (0.52)	0.182	4.37 (0.70)	4.63 (0.51)	0.014
Wave 2 (*n *= 233)	4.55 (0.60)	4.57 (0.61)	0.819	4.36 (0.72)	4.62 (0.55)	0.009
Life satisfaction (mean 0–10)						
Wave 2 (*n *= 231)	6.94 (1.98)	7.84 (1.75)	0.001	6.15 (2.06)	7.75 (1.70)	< 0.001

Supplementary material Appendix 1 presents descriptive data separately for survivors who reported both impaired wellbeing and achievements, and those who reported impaired performance only or impaired wellbeing only. These descriptive characteristics indicate that the symptom levels were particularly high among survivors with both perceived impaired performance and impaired wellbeing, whereas the symptom levels of those who reported impaired performance only, were more similar to those with unchanged and/or improved performance or wellbeing. To test if there was a non-linear relationship between exposure and impaired school functioning; e.g., that only the most severely exposed survivors would have impaired school functioning, we examined academic performance and school wellbeing according to four exposure categories based on the exposure scores for the 25th-, 50th-, and 75th-percentiles for our sample. However, impaired academic performance and impaired wellbeing at school were quite common also in survivors with exposure scores in the lower range of the scale (Supplementary material Appendix 2)

Table 3 displays study characteristics by MHS utilization. Compared to non-users, survivors who had used MHS were more likely to report either impaired or improved academic performance and school wellbeing. They were also more likely to have received auxiliary school support and to have needed practical facilitations. MHS utilization in the period 5–15 months after the attack (wave 2) was additionally associated with perceived needs for auxiliary academic assistance from teachers and additional social or emotional support. There were no significant differences with respect to satisfaction with the school support or completing the study year according to MHS utilization. In multivariate logistic regression analyses, higher age and posttraumatic stress reactions were associated with perceived impaired academic performance after additional adjustments for gender, somatic symptoms and social support (Table 4). When additionally adjusting for impaired school wellbeing (yes/no), age and impaired wellbeing were associated with impaired performance, but not posttraumatic stress reactions. With respect to impaired school wellbeing, only posttraumatic stress reactions remained associated with impaired wellbeing after similar adjustments. This association remained significant when additionally adjusting for impaired academic performance. In the post hoc analysis, non-Norwegian origin and being financially disadvantaged no longer remained significantly associated with impaired school wellbeing after adjusting for posttraumatic stress reactions, age and gender.

**Table 3:** Study characteristics for youth survivors the year following the terror attack according to mental health service (MHS) utilization approximately 0–5 months (wave 1) and 5–15 months (wave 2) after the attack

Characteristics	MHS utilization wave 1 (*n *= 207)		*p* value	MHS utilization wave 2 (*n *= 233)		*p* value
	Yes (*n *= 146)	No (*n *= 61)		Yes (*n *= 157)	No (*n *= 76)	
	*n* (%)	*n* (%)		*n* (%)	*n* (%)	
Academic performance (*n *= 233)						
Impaired	93 (65)	31 (51)	0.022	108 (70)	32 (43)	< 0.001
Unchanged	30 (21)	24 (39)		26 (17)	35 (47)	
Improved	21 (15)	6 (10)		20 (13)	8 (11)	
School wellbeing (*n *= 231)						
Impaired	46 (33)	10 (16)	0.005	52 (34)	13 (18)	0.01
Unchanged	62 (44)	42 (69)		66 (43)	47 (64)	
Improved	33 (23)	9 (15)		35 (23)	14 (19)	
Needed auxiliary school support						
(a) Practical support (*n *= 225)						
No/little	41 (30)	29 (48)	0.041	34 (23)	45 (61)	< 0.001
Some	31 (22)	12 (20)		35 (24)	15 (20)	
Much/very much	67 (48)	20 (33)		78 (53)	14 (19)	
(b) Academic support (*n *= 223)						
No/little	64 (47)	35 (57)	0.208	59 (41)	52 (70)	< 0.001
Some	27 (20)	13 (21)		36 (25)	10 (14)	
Much/very much	46 (34)	13 (21)		50 (34)	12 (16)	
(c) Social/emotional support (*n *= 222)						
No/little	84 (62)	45 (74)	0.249	83 (58)	62 (84)	< 0.001
Some	30 (22)	10 (16)		37 (26)	9 (12)	
Much/very much	22 (16)	6 (10)		24 (17)	3 (4)	
Received auxiliary school support (*n *= 230)						
Yes	96 (68)	32 (53)	0.034	113 (74)	34 (46)	< 0.001
No	45 (32)	29 (48)		39 (26)	40 (54)	
Satisfied with school support (*n *= 191)						
No/little	25 (21)	5 (11)	0.270	27 (21)	6 (11)	0.264
Some	23 (19)	12 (26)		26 (20)	13 (23)	
Much/very much	73 (60)	29 (63)		78 (60)	37 (66)	
Completed study year (*n *= 231)						
No	39 (27)	12 (20)	0.276	44 (29)	14 (19)	0.130
Yes	104 (73)	48 (80)		110 (72)	59 (81)

**Table 4:** Multivariate logistic regression analyses of survivor characteristics associated with impaired school functioning in the year following exposure to a terror attack

	Impaired academic performance (*n *= 233)						Impaired school wellbeing (*n *= 231)					
	Crude OR	95% CI	*p* value	Adjusted OR *n *= 209	95% CI	*p* value	Crude OR	95% CI	*p* value	Adjusted OR *n* = 206	95% CI	*p* value
Male gender	0.58	0.34–0.99	0.044	0.82	0.44–1.53	0.533	0.65	0.36–1.15	0.134	1.14	0.56–2.30	0.718
Age	1.06	0.95–1.18	0.300	1.18	1.03–1.36	0.015	0.98	0.88–1.10	0.766	1.07	0.94–1.22	0.296
Posttraumatic stress reactions	2.16	1.42–3.30	< 0.001	1.84	1.01–3.35	0.048	3.59	2.18–5.92	< 0.001	2.90	1.46–5.75	0.002
Somatic symptoms	2.88	1.58–5.26	0.001	1.81	0.79–4.12	0.159	3.78	2.04–7.02	< 0.001	1.58	0.68–3.66	0.285
Social support	0.70	0.42–1.18	0.185	0.59	0.65–2.15	0.592	0.49	0.29–0.82	0.006	0.87	0.48–1.56	0.638

Post hoc analysis	Impaired school wellbeing (*n *= 231)					
	Crude OR	95% CI	*p* value	Adjusted OR *n *=202	95% CI	*p* value
Male gender	0.65	0.36–1.15	0.134	1.13	0.55–2.31	0.738
Age	0.98	0.88–1.10	0.766	1.06	0.93–1.21	0.395
Posttraumatic stress reactions	3.59	2.18–5.92	< 0.001	3.54	1.99–6.29	< 0.001
Non-Norwegian origin	3.09	1.19–8.01	0.020	1.02	0.31–3.39	0.979
Financially disadvantaged	2.51	1.27–4.95	0.008	2.08	0.94–4.61	0.072

The outcomes, impaired academic performance and impaired school wellbeing were measured at wave 2, 14–15 months after the attack, whereas posttraumatic stress reactions, somatic symptoms and social support were measured at wave 1, 4–5 months after the attack

*OR* odds ratio, *CI* confidence interval

## Discussion

In the year following the terrorist attack, the majority of survivors perceived that their academic performance had worsened, with more than one in four reporting impaired school wellbeing. Furthermore, they declared substantial needs for school support. Our findings are in accordance with a recent register-based study of a smaller student sample of survivors from the Utøya attack, which displayed that their grades declined and their absence increased the year after the event [2].

Both impaired academic performance and impaired school wellbeing were associated with higher levels of mental and physical health problems, and lower life quality (Table 2). Most survivors with impaired wellbeing also reported impaired academic performance. Our findings suggest that survivors with impaired wellbeing at school the year following the attack were particularly at risk for poorer health and lower life quality (Appendix 1). Those who thrived less, reported significantly less social support. Furthermore, those who reported impaired academic performance and/or poorer school wellbeing were more likely to be less satisfied with the support they had received from their respective school/educational institutions. This relationship highlights the importance of promoting a supportive school environment in the aftermath of mass trauma. Prior research from war and disaster settings has demonstrated that school and teacher-based interventions could play a major role to promote children’s preparedness and resilience for coping with traumatic events, as well as to recover and regain normal routine [24, 25]. Survivors with poorer school performance and/or wellbeing were more likely to report higher levels of posttraumatic stress, symptoms of anxiety and depression and somatic symptoms both early in the study year (wave 1) and after the end of the year (wave 2). A recent meta-analysis documented that posttraumatic stress disorder had a particularly adverse impact on attention, verbal memory, and speed of information processing, which are all important for learning capacities [26]. Symptoms of posttraumatic stress, anxiety and depression as well as pain and other somatic symptoms can trouble concentration, sleep, and cognitive functioning. They may consequently adversely affect the academic performance and cause absence from school/studies. On the other hand, poorer achievements and impaired wellbeing at school may possibly also maintain, aggravate or generate psychological distress and/or psychosomatic ailments.

Survivors who disclosed impaired school functioning—both with respect to academic performance and wellbeing—were clearly more likely to report sleep problems both early in the school/study year (wave 1, 4–5 months after the attack) and after the first school year following the attack (wave 2, 14–15 months after the attack). Evaluation of sleep quality in a school/study setting may therefore be valuable to identify trauma-exposed youth at risk of impaired educational functioning and health adversity. With respect to sociodemographic factors, female survivors were more likely than male survivors to perceive their academic performance as impaired the year following the attack. Moreover, impaired wellbeing was more common in survivors who were financially disadvantaged and of non-Norwegian origin. However, after multivariate adjustments, the associations with sociodemographic variables no longer remained significant (Table 4). Whereas 29% of the survivors disclosed impaired wellbeing, 21% actually reported improved wellbeing at school/studies the year following the attack. We do not have data on why their wellbeing improved, but it is possible that they received both more formal and informal support at school/studies following the attack.

Those who used MHS were more likely to attain less and need more support at school than non-users. This could be expected due to higher levels of psychopathology in survivors who receive MHS [27].

Still, there were no significant differences with respect to satisfaction with support measures or self-reported completion of the study year by MHS utilization. This might indicate that MHS utilization facilitated access to support measures that could have helped survivors to function at school/studies despite posttraumatic stress reactions. An important question for future follow-up is how educational institutions and mental health professionals should work together to support youth in the wake of terrorist attacks and other mass trauma.

The close relationship between poor health, lower life quality and impaired school functioning underscores the importance of cooperation among school personnel and healthcare providers [28]. Our study indicates that assessment of school functioning is important to identify young survivors with a need for follow-up from school personnel and health care providers. Mental health professionals typically come into contact with trauma-exposed individuals through referral. Yet a major challenge in post-disaster outreach is to identify individuals in need of treatment. Indeed, prior research has uncovered high unmet needs in the aftermath of terrorist attacks [29, 30]. Teachers, school nurses and other educational staff may play a primordial role in primary prevention of post-trauma adversity and the identification of youth in need of treatment. The majority of the survivors reported impaired academic performance both at 4–5 months (69%) and 14–15 months (61%) after the attack. Hence, schools should evaluate the academic performance and organize educational support measures to prevent and/or improve impaired academic performance in trauma-exposed students. Although less prevalent, an important minority reported impaired school wellbeing at 4–5 months (30%) and 14–15 months (29%) after the attack. Such impairment in wellbeing was strongly associated with higher risk of mental health problems, lower life quality, and sleep problems. An important measure to strengthen the outreach to trauma-exposed youth may be to educate teachers about common posttraumatic stress reactions and behavioral changes they may observe after traumatic events. Furthermore, about how, when and to whom they should refer students in need of support measures. This study indicates that an assessment of impaired wellbeing at school and sleep problems, may be valuable for teachers and educational staff to identify students for referral to MHS.

There is a possibility of a bidirectional relationship between school functioning and mental health problems. Stress reactions may impair sleep, concentration and cognition, which may adversely impact school functioning. On the other hand, poorer academic performance and wellbeing at school and studies might also be psychologically burdensome and potentially maintain or worsen mental health problems. Early interventions might prevent the development of a vicious circle of accelerating mental health and educational problems. The school community may play a pivotal part in children’s and adolescents’ trauma recovery by promoting a supportive environment and a sense of belonging. School activities and routines may contribute to restore a feeling of normality and social equilibrium. Conversely, a lack of support, deterioration of academic achievement and negative reactions at school may prevent healing after trauma.

### Strengths and limitations

This study provides new knowledge on long-term school functioning and its relationship to health in young survivors of a terrorist attack. It provides new insight into the impact of single event trauma, whereas previous research primarily has addressed educational consequences of chronic forms of trauma through a cross-sectional assessment. Furthermore, all survivors in our study population were exposed to life-threatening events which have been identified. The relatively homogenous trauma exposure may have reduced the risk of confounding, and the clearly defined study population may have diminished the risk of selection bias. There were also several study limitations. Our study relied on self-reports, which may be inaccurate. Besides, we did not have comparison data from unexposed students in the general population. Moreover, the survivors went to different schools across geographical regions and levels; including lower and upper secondary schools, and higher education institutions. We lacked detailed data about how the respective schools supported survivors, and whether it differed between schools. Norway has a universal healthcare coverage, and public education is free of charge. Findings may not be directly generalizable to countries with a different organization of health services and education system. Future studies should explore in more depth the academic performance and wellbeing at school after exposure to traumatic events. The youths’ self-reports should be combined with objective data on school grades and absence, as well as reports from their teachers and parents with respect to their perception of how the youth cope and function following the traumatic event. Furthermore, a qualitative assessment may provide valuable insight into how the youth conceptualize wellbeing, and which factors that impact their wellbeing at school. There is also a need for research on the efficiency and efficacy of different types of school interventions in the aftermath of terrorist attacks and other mass trauma.

## Conclusion

A terrorist attack can have a considerable negative impact on young survivors’ academic performance and school wellbeing, which are further associated with poorer health. It is therefore essential to provide appropriate school support, and to coordinate the delivery of health care with proper follow-up at school.

## Electronic supplementary material

Below is the link to the electronic supplementary material.

Supplementary material 1 (XLSX 15 kb)

Supplementary material 2 (XLSX 11 kb)
